# DHEA use to improve likelihood of IVF/ICSI success in patients with diminished ovarian reserve: A systematic review and meta-analysis

**DOI:** 10.5935/1518-0557.20180046

**Published:** 2018

**Authors:** Juan Enrique Schwarze, Johana Canales, Javier Crosby, Carolina Ortega-Hrepich, Sonia Villa, Ricardo Pommer

**Affiliations:** 1 Reproductive Medicine Unit, Las Condes Clinic. Santiago, Chile; 2 Clinical Department of Obstetrics and Gynecology, Universidad de Santiago de Chile; 3 Reproductive Medicine Unit, Monteblanco Clinic. Santiago, Chile

**Keywords:** DHEA, diminished ovarian reserve, ICSI, IVF, poor ovarian response

## Abstract

The aim of this review is to determine if the use of DHEA increases the likelihood of success in patients with POR. We searched MEDLINE and EMBASE using the terms "DHEA and diminished ovarian reserve", "DHEA and poor response", "DHEA and premature ovarian aging". A fixed effects model was used and Peto's method to get the odds ratio (OR) with 95% confidence intervals (CI 95%). For quantitative variables, Cohen's method was used to present the standardized mean differences (SMD) with their corresponding confidence intervals. Only five studied fulfilled the selection criteria. DHEA was administered in 25 mg doses, three times a day. In all studies, the authors corrected for the presence of confounding variables such as partner's age, infertility diagnosis and number of transferred embryos. The meta-analysis of the five selected studies assessed a total of 910 patients, who underwent IVF/ICSI, of which 413 had received DHEA. DHEA use was associated with a significant increase in pregnancy likelihood (OR 1.8, CI 95% 1.29 to 2.51, *p*=0.001). When analyzing the association between DHEA use and the likelihood of abortion, we found low heterogeneity between studies (I^2^=0.0%) and the use of DHEA to be associated to a significant reduction in the likelihood of abortion (OR 0.25, CI 0.07 to 0.95; *p*=0.045). Analysis of the association of DHEA with average oocyte retrieval showed high variability between studies (I^2^=98.6%), as well as no association between DHEA use and the number of oocytes retrieved (SMD -0.01, CI 95% -0.16 to 0.13; *p*<0.05).

## INTRODUCTION

The management of patients with poor ovarian response (POR) with stimulation for *in vitro* fertilization (IVF) and intracytoplasmic sperm injection (ICSI) still poses great challenges. Retrieval of few oocytes is associated with a lower number of embryos for transfer and a lower success rate (Keay *et al.,* 1997). POR frequency is estimated at 5-18% for IVF/ICSI cycles, with a pregnancy rate as low as 2-4% (Yilmaz *et al*., 2013). POR is based on the presence of at least two of the following features: (i) older women, or any other POR risk factor; (ii) an earlier prior poor ovarian response; and (iii) an abnormal ovarian reserve test (low antral follicle count or low anti-Müllerian hormone (AMH) (Ferraretti *et al.,* 2011).

Different interventions have been tried on patients with POR, such as different stimulation protocols and adjuvant therapies to improve rates of ovarian response and pregnancy. Unfortunately, none of these regimes has been shown to be better over others (Pandian *et al*., 2010). The use of dehydroepiandrosterone (DHEA) prior to stimulation is one of these interventions. DHEA is an endogenous steroid, originating in the reticular zone of the suprarenal cortex and ovarian theca cells. It is an essential prohormone in ovarian follicular steroidogenesis (Burger, 2002; Casson *et al*., 2000). Casson *et al*. (2000) described the beneficial effects of DHEA supplements in ovarian stimulation in patients with POR. There is still speculation regarding its mechanism of action. Oral administration of DHEA increases serum levels of IGF-I, which ought to have a positive effect on follicular development and oocyte quality.

The aim of this review is to determine if the use of DHEA increases the likelihood of success in patients undergoing IVF/ICSI.

## MATERIALS AND METHODS

We searched MEDLINE and EMBASE using the terms "DHEA and diminished ovarian reserve", "DHEA and poor response", "DHEA and premature ovarian aging". Articles were first selected by title and abstract and then full text copies were obtained and scrutinized following our selection criteria. Two authors (JES and JC) revised the results from the search independently and retrieved those articles that fulfilled the selection criteria. In case of disagreement, a third author acted as referee. A reference list of relevant articles was also searched manually, looking for additional studies. We included studies comparing pregnancy rates in patients undergoing IVF/ICSI, in patients who received DHEA prior to ovarian stimulation, published between 2007 and 2017 either in English or in Spanish. We included studies that considered POR by the presence of at least two of the three following criteria: (a) patients older than 40 years of age; (b) antral follicle count lower than 5, or decreased AMH; (c) a deficient prior ovarian response. Secondary studies (i.e. systematic reviews, meta-analyses), and studies in which an additional drug was administered in conjunction with DHEA were excluded.

The collated data was transferred to a proforma form including: references, year of publication, inclusion criteria, type of study, DHEA doses, number of patients, number of clinical pregnancies, number of abortions and mean oocyte retrieval. The primary objective was the clinical pregnancy rate per initiated cycle. Clinical pregnancy was considered as the identification of at least one embryo displaying cardiac activity. Secondary objectives were average oocyte retrieval and abortion frequency.

We used STATA (STATA Corp. USA) for the meta-analysis. The heterogeneity between studies was tested using the Chi-squared test. For qualitative variables, a fixed effects model was used and Peto's method to get the odds ratio (OR) with 95% confidence intervals (CI 95%). For quantitative variables, Cohen's method was used to present the standardized mean differences (SMD) with their corresponding confidence intervals. The results were recorded in a forest plot.

## RESULTS

**Figure 1:**
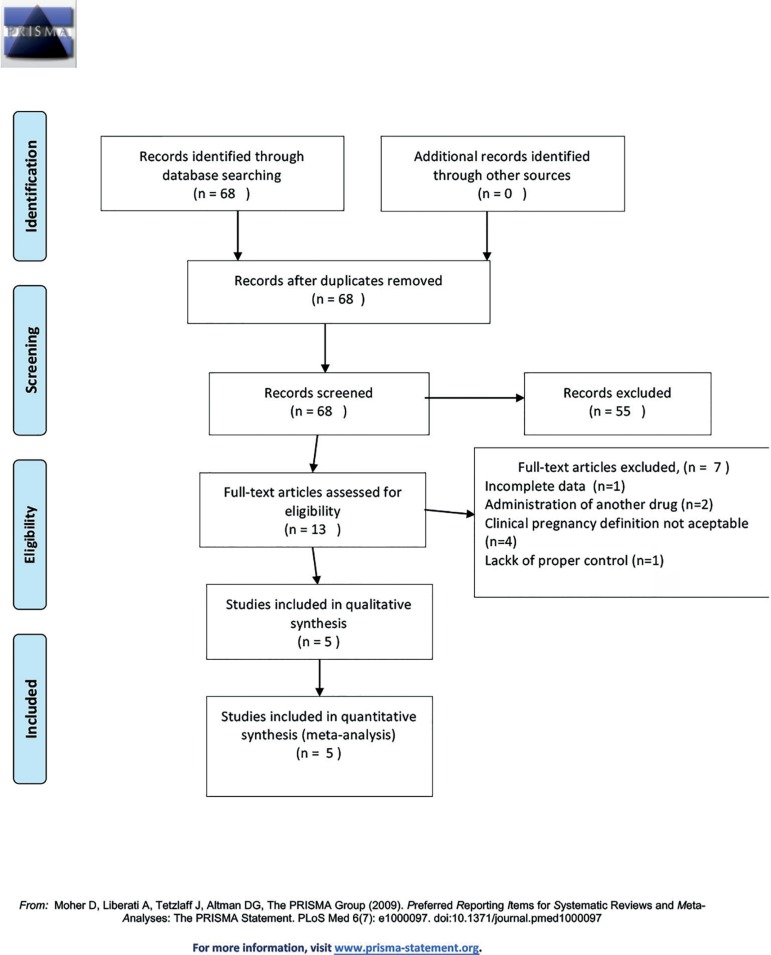
Flow Diagram.

We initially identified 68 potentially relevant studies. After reading all the abstracts, 55 studies were excluded and full copies of the 13 remaining studies were retrieved. Only five studied fulfilled the selection criteria. Figure 1 shows the reasons for exclusion of studies. Studies included in this systematic review were published between 2007 and 2016 and their various study designs included: two retrospective studies (Barad *et al.,* 2007; Xu *et al.,* 2014), two randomized clinical trials (Wiser *et al*., 2010; Kotb *et al*., 2016), and one prospective cohort study (Vlahos *et al.*, 2015). In all of these studies, DHEA was administered in 25 mg doses, three times a day. DHEA courses prior to IVF/ICSI cycles were: six weeks (Wiser *et al*., 2010); 12 weeks (Xu *et al.,* 2014; Kotb *et al*., 2016; Vlahos *et al.*, 2015); and 16 weeks (Barad & Gleicher, 2006). Four studies reported the use of DHEA to be associated with an increase in pregnancy rate (Xu *et al.,* 2014; Wiser *et al*., 2010; Kotb *et al*., 2016; Barad & Gleicher, 2006) whereas one study found a decrease (Vlahos *et al.*, 2015). In four studies, average oocyte retrieval was higher in groups receiving DHEA (Xu *et al.,* 2014; Wiser *et al*., 2010; Kotb *et al*., 2016; Vlahos *et al.*, 2015) whereas one study recorded a lower average (Barad & Gleicher, 2006) (Table 1).

**Table 1:** Summary of studies included

Study	Type of study	Outcome	Inclusion criteria	Exposure	Pregnancies
Barad *et al*., 2007	Case control	Mean number of oocytes recovery Implantation rate Clinical Pregnancy rate Abortion rate	FSH >12mIU/ml E_2_≥75pg/ml	25mg tid for 4 months	DHEA group: 13/64 pregnancies Control group: 11/101 pregnancies
Wiser *et al*., 2010	Randomized controlled trial	Mean number of oocytes recovery Clinical Pregnancy rate Abortion rate	Previous IVF cycle with more than 300IU rFSH/day Less than 5 embryos	25mg tid for 6 weeks	DHEA group: 04/16 pregnancies Control group: 2/16 pregnancies
Xu *et al*., E22014	Retrospective	Implantation rate Clinical Pregnancy rate	Two or more of following: ≥40years <4 oocytes recovered in previous cycle <5AFC	25mg tid for 90 days	DHEA group: 57/189 pregnancies Control group :37/197 pregnancies
Vlahos *et al*., 2015	Prospective	Clinical Pregnancy rate Delivery of a live born	Two of following: ≥40years <3 oocytes recovered in previous cycle E2 peak <500pg/ml	25mg tid for 12 weeks	DHEA group: 1/48 pregnancies in Control group 8/113 pregnancies
Kotb *et al*., 2016	Randomized controlled trial	Clinical Pregnancy rate	Bologna criteria	25mg tid for 3 months	DHEA group 20/70 pregnancies Control group: 9/70 pregnancies

Only three studies recorded abortion rates and in all of them the rates were lower in those groups that had been given DHEA (Wiser *et al*., 2010; Vlahos *et al.*, 2015; Barad & Gleicher, 2006). In all studies, the authors corrected for the presence of confounding variables such as partner's age, infertility diagnosis and number of transferred embryos (Xu *et al.,* 2014; Wiser *et al*., 2010; Kotb *et al*., 2016; Vlahos *et al.*, 2015; Barad & Gleiche, 2006).

**Figure 2:**
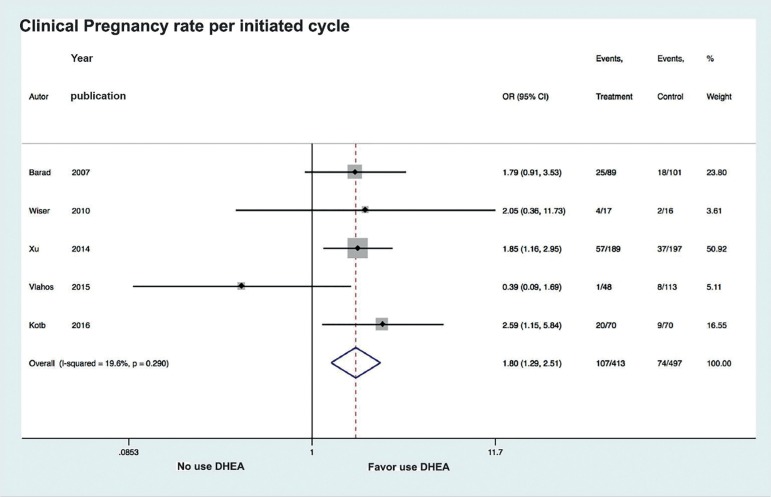
Forest plot of comparison: DHEA versus control, outcome: Clinical Pregnancy rate per initiated cycle.

**Figure 3:**
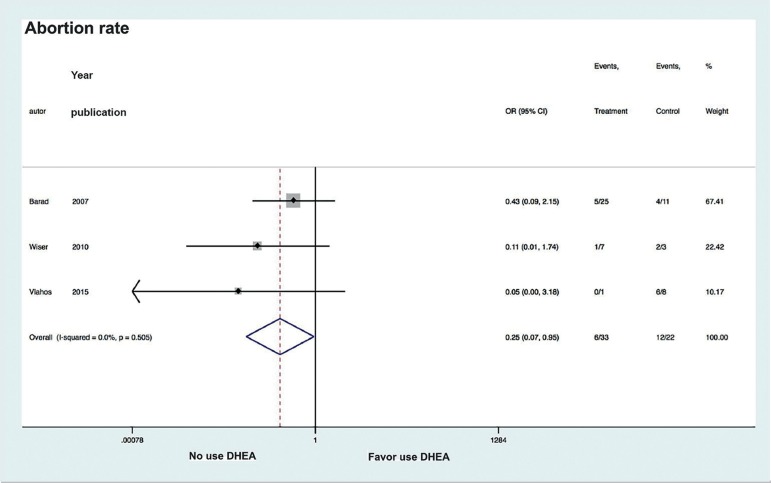
Forest plot of comparison: DHEA versus control, outcome: Abortion rate.

**Figure 4:**
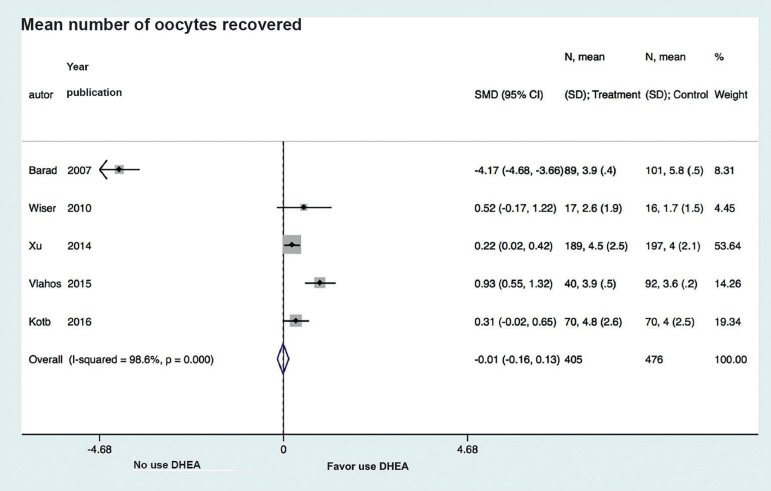
Forest plot of comparison: DHEA versus control, outcome: Mean number of oocytes recovered.

The meta-analysis of the five selected studies assessed a total of 910 patients who underwent IVF/ICSI, of which 413 had received DHEA. Analysis of the association between DHEA and likelihood of pregnancy revealed low heterogeneity between studies (I^2^=19.6%). DHEA use was associated with a significant increase in pregnancy likelihood (OR 1.8, CI 95% 1.29 to 2.51, *p*=0.001) (Figure 2). When analyzing the association between DHEA use and likelihood of abortion, we found low heterogeneity between studies (I^2^=0.0%), and the use of DHEA to be associated to a significant reduction in the likelihood of abortion (OR 0.25, CI 0.07 to 0.95; *p*=0.045) (Figure 3). Analysis of DHEA association with average oocyte retrieval showed high variability between studies (I^2^=98.6%) as well as no association between DHEA use and the number of oocytes retrieved (SMD -0.01, CI 95% -0.16 to 0.13; *p*<0.05) (Figure 4).

## DISCUSSION

This review evaluated the effects of DHEA treatment on IVF/ICSI outcomes in patients with POR. Our findings indicate that the use of DHEA is associated with a better pregnancy rate, a lower frequency of abortion, but without affecting average oocyte retrieval.

Our results differ from a previous meta-analysis carried out by Narkwichean *et al*., in which three studies, between 1980 and 2012, were analyzed and the authors found no association of treatment with DHEA with an improvement neither in the average oocyte retrieval, nor in the number of pregnancies (Narkwichean *et al*., 2013). Our study also did not find a difference in the average oocyte retrieval, but it did show an improvement in the clinical pregnancy rate. This implies that an improved clinical pregnancy rate might be due to an improvement in oocyte quality. Our findings of a lower rate of abortion in the DHEA groups further support this.

A potential limitation for this meta-analysis may be the fact that stimulation protocols differed between studies. However, there was no difference within each study in stimulation protocols between patients who received DHEA and those who did not. Another possible cause for confusion might be the difference in the number of weeks of DHEA administration, which varied between six and 14 weeks. However, the likelihood of pregnancy improved irrespective of the number of weeks DHEA was administered.

The mechanism of action whereby DHEA use might improve oocyte quality and or endometrium receptivity is yet to be elucidated, which is clinically translated into an improved pregnancy rate and a decrease in the abortion rate. In addition, it is yet to be determined whether there are differences between the different POR profiles (previous poor ovarian response age, or altered ovarian reserve tests) and DHEA use prior to ovarian stimulation.

We found that the use of DHEA prior to ovarian stimulation in women with POR is associated with an improvement in prognosis. Taking into account the outcomes of this review and given that DHEA is a well-tolerated drug; our recommendation is that DHEA should be included in the treatment of patients with POR.

## References

[r1] Barad D, Gleicher N (2006). Effect of dehydroepiandrosterone on oocyte and embryo yields,
embryo grade and cell number in IVF. Hum Reprod.

[r2] Barad D, Brill H, Gleicher N (2007). Update on the use of dehydroepiandrosterone supplementation among
women with diminished ovarian function. J Assist Reprod Genet.

[r3] Burger HG (2002). Androgen production in women. Fertil Steril.

[r4] Casson PR, Lindsay MS, Pisarska MD, Carson SA, Buster JE (2000). Dehydroepiandrosterone supplementation augments ovarian
stimulation in poor responders: a case series. Hum Reprod.

[r5] Ferraretti AP, La Marca A, Fauser BC, Tarlatzis B, Nargund G, Gianaroli L (2011). ESHRE working group on Poor Ovarian Response Definition. ESHRE
consensus on the definition of 'poor response' to ovarian stimulation for in
vitro fertilization: the Bologna criteria. Hum Reprod.

[r6] Keay SD, Liversedge NH, Mathur RS, Jenkins JM (1997). Assisted conception following poor ovarian response to
gonadotrophin stimulation. Br J Obstet Gynaecol.

[r7] Kotb MM, Hassan AM, AwadAllah AM (2016). Does dehydroepiandrosterone improve pregnancy rate in women
undergoing IVF/ICSI with expected poor ovarian response according to the
Bologna criteria? A randomized controlled trial. Eur J Obstet Gynecol Reprod Biol.

[r8] Narkwichean A, Maalouf W, Campbell BK, Jayaprakasan K (2013). Efficacy of dehydroepiandrosterone to improve ovarian response in
women with diminished ovarian reserve: a meta-analysis. Reprod Biol Endocrinol.

[r9] Pandian Z, McTavish AR, Aucott L, Hamilton MP, Bhattacharya S (2010). Interventions for 'poor responders' to controlled ovarian hyper
stimulation (COH) in in-vitro fertilisation (IVF). Cochrane Database Syst Rev.

[r10] Vlahos N, Papalouka M, Triantafyllidou O, Vlachos A, Vakas P, Grimbizis G, Creatsas G, Zikopoulos K (2015). Dehydroepiandrosterone administration before IVF in poor
responders: a prospective cohort study. Reprod Biomed Online.

[r11] Wiser A, Gonen O, Ghetler Y, Shavit T, Berkovitz A, Shulman A (2010). Addition of dehydroepiandrosterone (DHEA) for poor-responder
patients before and during IVF treatment improves the pregnancy rate: a
randomized prospective study. Hum Reprod.

[r12] Yilmaz N, Uygur D, Inal H, Gorkem U, Cicek N, Mollamahmutoglu L (2013). Dehydroepiandrosterone supplementation improves predictive
markers for diminished ovarian reserve: serum AMH, inhibin B and antral
follicle count. Eur J Obstet Gynecol Reprod Biol.

[r13] Xu B, Li Z, Yue J, Jin L, Li Y, Ai J, Zhang H, Zhu G (2014). Effect of dehydroepiandrosterone administration in patients with
poor ovarian response according to the Bologna criteria. PLoS One.

